# A rare case of a breast hamartoma containing a lesion with malignant presentation on radiologic evaluation and benign presentation on pathologic findings: A case report

**DOI:** 10.1016/j.radcr.2024.10.037

**Published:** 2024-10-23

**Authors:** Kiarash Soltani, Mahdi Taghdiri, Elham Keshavarz, Saloomeh Mohammadi

**Affiliations:** a School of medicine, Shahid Beheshti University of Medical Sciences, Tehran, Iran; b Department of radiology, Tehran University of Medical Sciences, Imam Khomeini hospital, Tehran, Iran; c Department of radiology, Mahdiyeh hospital, Shahid Beheshti University of Medical Sciences, Tehran, Iran; d Department of pathology, Mahdiyeh hospital، Shahid Beheshti University of Medical Sciences, Tehran, Iran

**Keywords:** Breast, Hamartoma, Invasive carcinoma, BIRADS

## Abstract

Mammary hamartomas are rare, benign, and often under-diagnosed breast lesions comprised of glandular, adipose, muscular, and fibrotic encapsulated components which occur more commonly among perimenopausal women. The term hamartoma was initially coined by Arrigoni in 1971 to describe breast lesions with clinical resemblance to fibroadenomas. The proper diagnosis of breast hamartoma is confirmed through using multiple radiologic and pathologic modalities. Hamartomas rarely progress into malignancy with only a few cases reported in the literature. Here, we report a case of a highly suspicious lesion developing within a breast hamartoma in a 46-year-old woman with radiologic features of a malignant lesion with benign pathology.

## Introduction

Breast hamartomas are rare, benign, and encapsulated lesions of unknown cause and pathogenesis [1,2]. These lesions may be comprised of glandular, adipose, muscular, and fibrotic tissue often with heterogenous tissue distribution [3,4]. Hamartomas rarely progress into malignant tissue, mainly due to similar composition to normal breast tissue [2]. With an overall low prevalence and female predominance, these lesions are being more frequently diagnosed mainly due to widespread breast cancer screening programs [5]. Notably, hamartomas can often be misdiagnosed as fibroadenoma which means that their prevalence may actually be higher [6,7]. The proper diagnosis of breast hamartoma is confirmed through using multiple diagnostic radiologic and pathologic modalities such as mammography, ultrasonography, magnetic resonance imaging, fine-needle aspiration biopsy, and core biopsy [2,8,9]. It is worth mentioning that due to lack of distinctive cytological and histological features, clinical diagnosis of hamartomas requires a multimodal approach [2]. Surgical excision is the treatment of choice in the majority of the hamartoma cases [10,11]. Herein, we report a rare case of a breast hamartoma containing a highly suspicious mass with pathologic and radiologic discordancy.

## Case presentation

A 46-year-old woman was referred to Mahdiyeh hospital, a university-affiliated hospital located in Tehran, Iran in August 2024 concerning the follow-up of a mass located in the left breast of the patient. The patient claimed that the mass has been present there for many years and cannot remember the exact time when she was first aware of the mass. Family history was unremarkable. No history of Tabacco use was reported by the patient. Physical examination revealed a painless, soft, mobile, and palpable mass in the outer (lateral) part of the left breast.

**Fig. 1:**
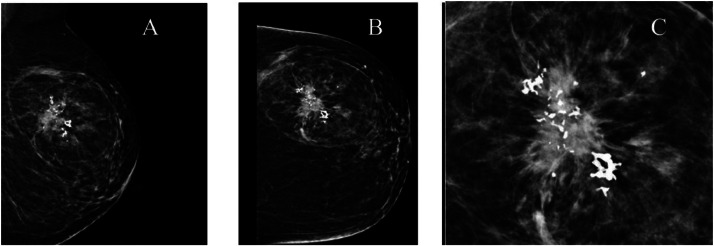
(A and B) Mediolateral Oblique (MLO) View and Craniocaudal (CC) View mammograms of left breast. An oval circumscribed, predominantly fatty mass in the left breast Upper Outer Quadrant (UOQ). (C) magnified view of the lesion indicates a hyperdense mass with irregular shape and spiculated margin associated with coarse heterogeneous microcalcifications within it.

Bilateral mammography showed an oval shaped circumscribed predominantly fatty mass measuring about 9.5 × 7.5 × 8 cm in the upper outer quadrant of the left breast (Fig. 1A, and B). In addition, a hyperdense mass measuring approximately 4.5 × 4 × 3 cm with irregular shape and spiculated margins associated with coarse heterogenous microcalcifications was seen within the mentioned lesion (Fig. 1C). BI-RADS V score as given.

**Fig. 2:**
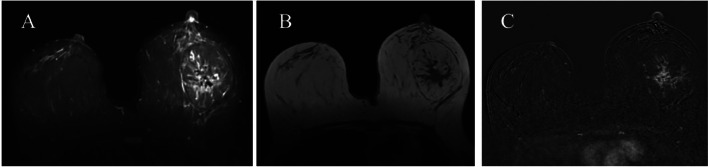
(A) Axial Turbo inversion recovery magnitude (TIRM), (B) Axial T1 fast spin echo, (C) Axial subtraction post contrast phase. An oval circumscribed mass of predominant internal fat intensity with an internal irregular spiculated mass of heterogenous T2 hypo intensity, heterogenous enhancement and medium/plateau kinetics.

Due to the unusual presentation of the mass and its relatively large size, magnetic resonance imaging (MRI) evaluation was ordered. MRI evaluation confirmed the presence of an oval shaped circumscribed mass of predominant internal adipose intensity (nonenhancing) containing an internal irregular spiculated mass of heterogenous T2 hypo-intensity with heterogenous enhancement and medium/plateau kinetics (Fig. 2). BI-RADS V score was again confirmed.

**Fig. 3:**
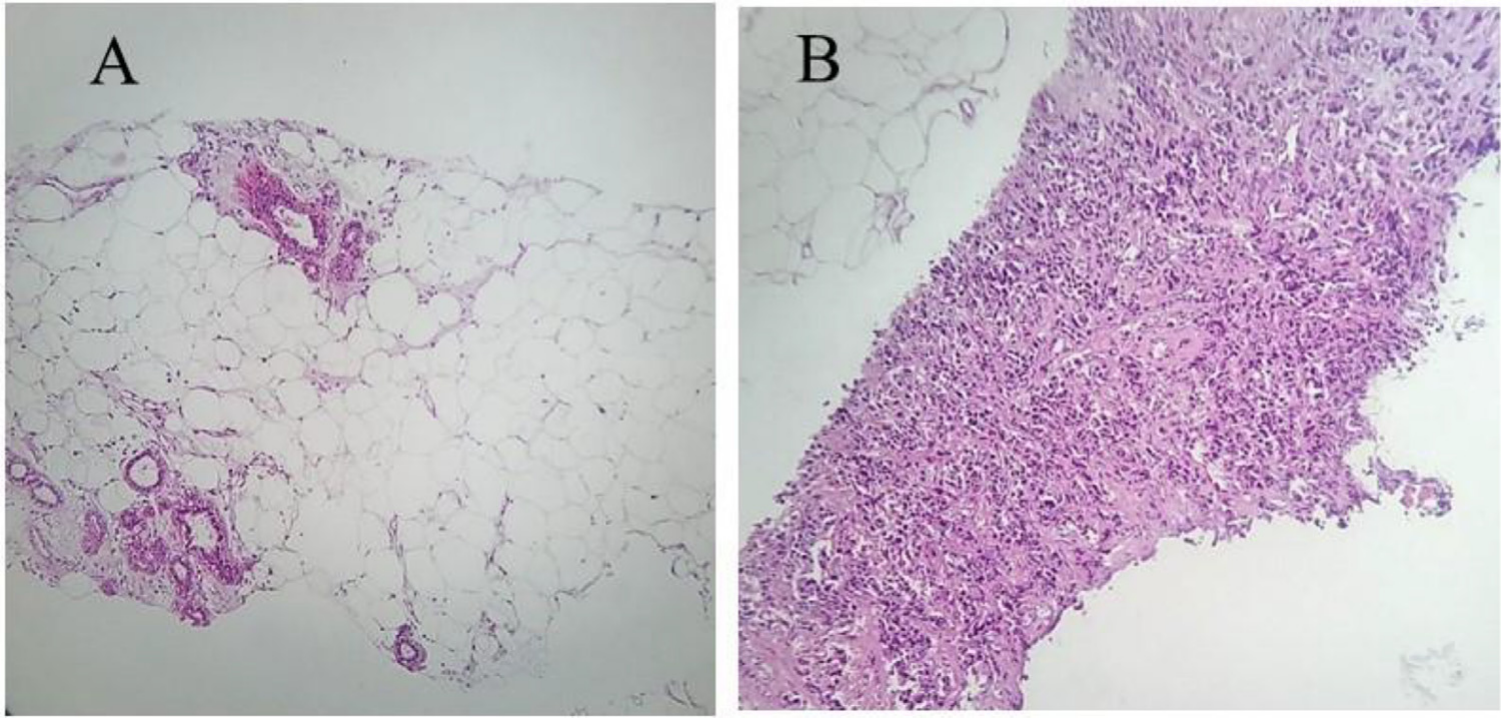
(A) Histopathologic view of the ultrasound guided biopsy specimen. Lobules and ducts of the mammary gland, and fibrous stroma and adipose tissue are located in a disorganized structure. Glands appear structurally normal with little proliferation. Neither cytoarchitectural atypia, nor significant mitosis is observed. (B) a view of invasive ductal carcinoma showing tumor clusters with focal tubule formation with desmoplastic stroma (the picture is for comparison and is not related to our patient). In contrast, invasive ductal carcinoma mostly has irregular borders and is comprised of atypical cells forming ducts and sheets with an infiltrative pattern in a desmoplastic stroma. Nuclear pleomorphism is evident, necrosis, and mitosis are also have been observed.

Upon pathologic evaluation, ultrasound-guided core needle biopsy was obtained from the irregular mass with revealed a benign mass comprised of ducts, lobules, and adipose tissue with disorganized structure (Fig. 3).

**Fig. 4:**
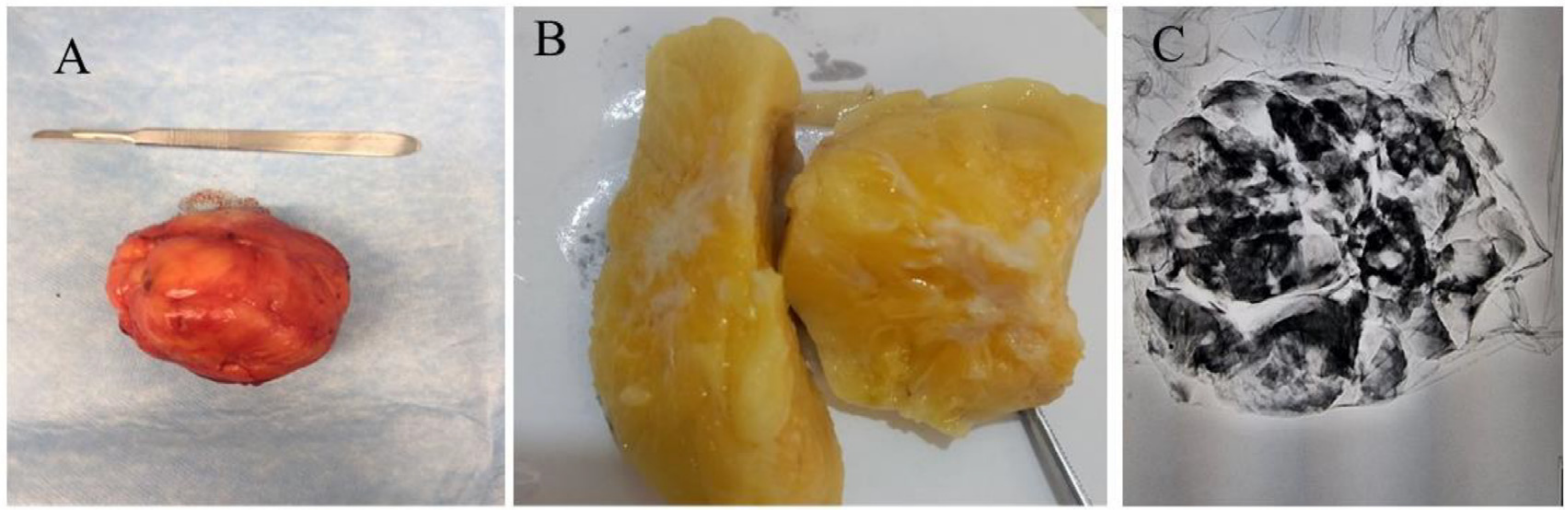
(A) Postsurgical resection specimen (Gross pathology), (B) inside the resected tumor was yellow and white with areas of fat and calcifications, (C) mammographic view of the resected tumor.

Due to the discordancy in radiologic and pathologic findings, and after the case was discussed in our multidisciplinary team, the patient underwent surgery and left breast mass was excised. On gross examination, the mass appears as a benign, well-circumscribed breast tumor composed of randomly arranged and well-differentiated glandular and stromal mammary structures, and also adipose tissue and smooth muscles (Fig. 4). The histologic evaluation of lobules and ducts within the adipose tissue was normal. The microscopic evaluation of the excised mass also revealed that lobules and ducts of the mammary gland, and fibrous stroma and adipose tissue are placed in a disorganized structure. Mammary glands appear structurally normal with little proliferation. Based on our evaluations, the patient had normal glands and lobules, and also neither cytoarchitectural atypia, nor significant mitosis were observed. Histopathologic findings also confirmed the core biopsy results.

## Discussion

Hamartomas are well-circumscribed, benign, and slow-growing masses containing varying degrees of fibrous tissue, adipose tissue, glandular tissue, and to some extent muscular tissue [1,12]. The average size of hamartomas is approximately 2-5 cm in diameter, however larger sizes have also been reported [13]. These lesions are rarely detected among men and usually occur in middle-aged, perimenopausal women accounting for less than 1 percent of all benign breast lesions [5,14].

Histologically, these lesions are composed of acinar units, arranged in a lobular pattern within a hyalinized, fibrous stroma and contain variable amounts of fat content in the interlobular regions [15,16].

The characteristic mammographic appearance of a hamartoma consists of a circumscribed mass with a thin pseudo capsule and a mix of fatty and soft-tissue elements [17]. The mammographic density of the mass can vary depending on the fat—parenchyma ratio. Although lesion with high fat content can mimic a lipoma, lesions with a predominance of glandular or fibrous tissues may be confused with other solid masses [[18], [19], [20]].

On MRI evaluation, T1- and T2-weighted sequences, the mass generally exhibits heterogeneous signal intensity, reflecting the presence of glandular and adipose tissue components contained within a thin capsule. After administration of contrast medium, hamartomas show a gradual, progressive enhancement with a type I time/intensity curve. By providing a cross-sectional view of the lesion, breast MRI is considered a more advantageous imaging technique compared to mammography or ultrasound in confirming the diagnosis of hamartoma [[20], [21], [22], [23]]. Hamartomas are challenging to diagnose, therefore, the use of a variety of breast screening modalities is warranted such as biopsy and different imaging techniques.

On gross examination, hamartomas are well-defined masses with smooth glistening surfaces when cut. Microscopically, they are composed of a combination of epithelial and stromal elements, usually with normal ducts or lobules, although variations of normal tissue may also be present [[24], [25], [26]].

Breast hamartomas are not considered premalignant lesions and are generally a benign lesion with rare presentation of malignancy. The probability of normal breast tissue within hamartoma to undergo malignant changes is as low as 0.1%. It is important to note that cases of carcinoma with benign histology have been reported within or adjacent to hamartomas and the possibility of malignancy should not be neglected [2,24,[27], [28], [29]].

In conclusion, we report a rare case of hamartoma in which different radiologic findings were in favor of a highly malignant tissue growing within the hamartoma. Subsequent evaluations revealed otherwise, and malignancy was ruled out. We believe that this presentation is extremely rare and given the low number of previous case reports regarding hamartomas our case is interesting to be examined.

## Patient consent

Written informed consent has been obtained from the patient in Persian (the patient's native language). Upon request, we will send it to the respected journal.
